# Fucose-containing fraction of Ling-Zhi enhances lipid rafts-dependent ubiquitination of TGFβ receptor degradation and attenuates breast cancer tumorigenesis

**DOI:** 10.1038/srep36563

**Published:** 2016-11-10

**Authors:** Shu-Ming Tsao, Hsien-Yeh Hsu

**Affiliations:** 1 Department of Biotechnology and Laboratory Science in Medicine, National Yang-Ming University, Taipei, Taiwan; 2 The Genomics Research Center, Academia Sinica, Taipei, Taiwan; 3 Program in Molecular Medicine, National Yang-Ming University and Academia Sinica, Taipei, Taiwan

## Abstract

*Ganoderma lucidum* exerts antitumor activity, but the mechanism of *G. lucidum* polysaccharides on cancer is unclear. Here, we demonstrated that a fucose-containing fraction of Ling-Zhi (FFLZ) reduced tumor size and suppressed metastasis *in vivo*. Furthermore, FFLZ inhibited breast cancer cell migration and altered the epithelial-to-mesenchymal transition (EMT) phenotype. Transforming growth factor-β receptor (TGFR) pathways act as key mediators to promote tumor progression and metastasis. We found that FFLZ down-regulated TGFR and downstream signaling pathways, including the phosphorylation of Smad2/3 and the expression of Smad4. In an investigation of the underlying mechanisms, we found that FFLZ enhanced the Smurf2-dependent ubiquitination of TGFR by disrupting the balance of the lipid rafts, promoted the “re-localization” of the TGFR to the caveolae, and facilitated the degradation of TGFR. Together, our data indicated that FFLZ is associated with the inhibition of EMT and the prevention of metastasis by promoting ubiquitination-dependent TGFR degradation and abolishing TGFR signaling pathways. Moreover, the combination of FFLZ and trastuzumab synergistically inhibited the viability of certain trastuzumab-resistant human breast cancer cells. In summary, our current findings indicate that FFLZ is a potential therapeutic or dietary supplemental agent for cancer patients and that it functions via the caveolin-1/Smad7/Smurf2-dependent ubiquitin-mediated degradation of TGFR.

*Ganoderma lucidum* (a medicinal fungus, aka Ling-Zhi in Chinese) has been used as a traditional Asian medicine to promote good health and longevity^1^. Previously, we reported that an active fucose-containing glycoprotein fraction isolated from a water-soluble Ling-Zhi extract, i.e., FFLZ, exerted antibody-mediated cytotoxicity and reduced the production of tumor-associated inflammatory mediators^2^, as well as exhibiting significant antitumor activity and immunological functions^1^. However, the molecular mechanism associated with the anti-metastatic activity of FFLZ in breast cancer is less clear and should be further investigated.

Breast cancer is among the most common malignant diseases. More than 90% of breast cancer-related deaths are caused by metastasis, not primary tumors^3^,^4^. Recent findings indicate that the inhibition of the activity of transforming growth factor-β1 (TGFβ) and/or TGFβ receptors (TGFR) enhances the action of chemotherapy against triple-negative breast cancer^5^. TGFβ, a multifunctional cytokine, is found in various cell types, with functions including cell proliferation, migration, and invasion in cancer^6^. Indeed, TGFβ is highly up-regulated in late-stage breast cancer^6^,^7^,^8^. Interestingly, TGFβ acts to induce tumor progression and metastasis during the late stages of breast carcinogenesis^9^,^10^,^11^ by the Smad and non-Smad phosphatidylinositol–3-kinase/AKT signaling pathways^12^. TGFβ can be described as a tumor promoter; one of its abilities is the induction of the TGFβ-mediated epithelial-to-mesenchymal transition (EMT), an important metastatic process in which epithelial cells convert to a mesenchymal cell phenotype^13^,^14^.

It has been reported that the level of TGFRs is regulated by ubiquitin-dependent proteasomal pathways (UPPs)^15^. E3 ubiquitin ligase Smurf2 (Smad ubiquitination regulatory factor 2) participates in modulating TGFβ-mediated signaling by targeting the ubiquitination of TGFR^16^. Unlike growth factor receptors that directly recruit E3 ligases, TGFRI requires an adaptor protein, Smad7, to recruit its E3 ligase^17^. In addition, Smad7 stabilizes the Smurf2-TGFRI complex and help E3 ligase-Smurf2 to ubiquitylate TGFRI. Furthermore, the trafficking of TGFRs is intimately linked to the control of the activity and termination of signaling events. A two-step regulation of TGFR has been proposed: in the first step, TGFR perform trafficking via the clathrin-mediated or lipid rafts/caveolae-mediated pathways to activate or inhibit signaling. Specifically, the clathrin-dependent internalization of TGFR is followed by the promotion of signal transduction^18^; alternatively, the lipid rafts/caveolae-dependent pathway attenuates TGFR signaling by enhancing the UPP of TGFRI. In the second step, during ubiquitin attachment, TGFRs are internalized by the proteasome complex via an endocytosis-mediated pathway^18^. A scheme summarizing the proposed FFLZ functions is presented in Sup. Fig. 5.

It is important that the inhibition of TGFβ and/or TGFR activity enhances the action of chemotherapy against triple-negative breast cancer^5^. It has also been reported that TGFβ is associated with cell resistance to trastuzumab and cooperates with HER2 through both Smad-dependent and -independent mechanisms^19^. Furthermore, a significant number of patients with triple-negative cancer or trastuzumab-resistant cancer do not benefit from targeted therapy with trastuzumab^20^. Hence, our study sought to elucidate the mechanisms by which FFLZ enhances TGFR degradation in relation to reductions in tumor proliferation and the synergistic effects of FFLZ and trastuzumab.

Altogether, our current findings indicate that FFLZ inhibits the viability of cancer cells and reduces breast tumorigenesis. Moreover, FFLZ inhibits migration during the EMT by suppressing TGFR-mediated signaling via the lipid rafts/caveolae-mediated ubiquitin-dependent degradation of TGFR. Furthermore, our research revealed that the combination of trastuzumab and FFLZ exhibits a synergetic antitumor effect in trastuzumab-resistant and/or triple-negative breast cancer cells, suggesting that this combination might provide a novel regimen for clinical breast cancer treatment.

## Results

### Fucose-containing fraction of Ling-Zhi (FFLZ) inhibits carcinogenesis in 4T1 breast cancer-bearing BALB/c mice *in vivo*

**Figure 1:**
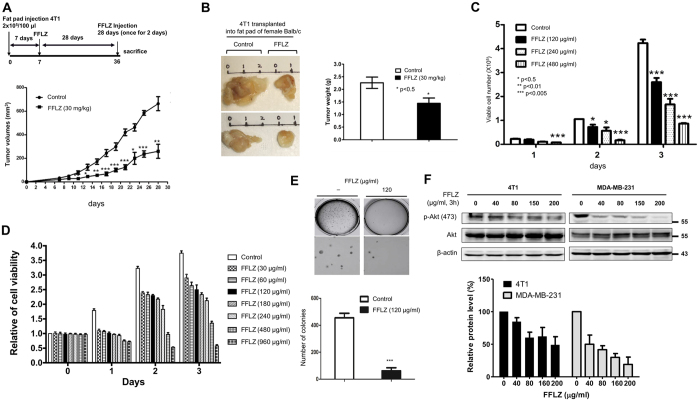
Fucose-containing fraction of Ling-Zhi (FFLZ) inhibits carcinogenesis *in vivo* and *in vitro*. (**A**) FFLZ inhibits tumor growth in 4T1-bearing mice. 4T1 cells were implanted into mice mammary fat pad tissue (n = 6 in each group). After 7 days, mice were treated with FFLZ (30 mg/kg) i.p. injection at intervals of 2 days for 28 days. Data shown are the mean ± standard deviation from three independent experiments. (**B**) Image and tumor weight of 4T1 transplanted into fat pads of female BALB/c mice. (*p < 0.05, **p < 0.01, ***p < 0.001; log-rank test for significance). (**C**) 4T1 cells (1 × 10^5^/well in 12-well plate) were treated with various dosages of FFLZ (120–480 μg/ml) for 1–3 days as indicated. The viability of cells was determined by cell counting assay. (**D**) 4T1 cells (1 × 10^4^/well in 96-well plate) were treated with various dosages of FFLZ (30–960 μg/ml) for 1–3 days as indicated. Cell viability of cells was determined by the MTT assay. Each group at 30–960 μg/ml of FFLZ is normalized to each un-treated control. The data are representative of three separate experiments with mean ± SD; error bars indicate SD. (**E**) Approximately 3,000 cells were seeded into each well of a six-well plate. The wells were filled with melted 0.33% agar solution, followed by treatment with FFLZ (120 μg/ml) for 26 days. The cell numbers of the colonies formed were determined, and the values shown represent the means ± SD, *p < 0.05, and quantitation of colony formation ability. (**F**) FFLZ decreases phosphorylation of Akt (a TGFR-non-Smad signaling pathway). 4T1 and MDA-MB-231 cells (1 × 10^5^ cells/ml) were treated with FFLZ (0–200 μg/ml) for 3 h. Total cell lysates were collected after the indicated incubation times and analyzed by western blot assays for phosphorylated AKT. Relative quantification analysis of phosphorylation-AKT band intensities by ImageJ. Representing one of three separate determinations in same experimental conditions.

Using 4T1-bearing mice, we demonstrated that FFLZ reduces tumor growth *in vivo*. Briefly, after the subcutaneous (s.c.) transplantation of 4T1 breast cancer cells into mice, along with concurrent FFLZ treatment, we found that FFLZ significantly suppressed 4T1 cell growth (Sup. Fig. 1A), reduced tumor weight (FFLZ group *vs*. control group) (Sup. Fig. 1B), and prolonged the survival rate of tumor-bearing mice (Sup. Fig. 1C). Next, the effect of FFLZ on mouse mammary fat pads injected with 4T1 cells was examined. Specifically, we injected 4T1 cells s.c. into the mammary fat pads of Balb/c mice, followed by FFLZ treatment, as in Fig. 1A. The mice were sacrificed on day 28, when the tumor volume had reached approximately 700 mm^3^ (control group). We found that the tumor volume (Fig. 1A, 610 ± 18 mm^3^ *vs*. 180 ± 20 mm^3^) and tumor weight (Fig. 1B, 2.3 ± 0.2 g vs. 1.45 ± 0.15 g) in the FFLZ-treated 4T1-bearing mice were significantly decreased compared to the control mice with PBS treatment. In contrast, no apparent difference in body weight was observed between the FFLZ-treated mice and the PBS-treated control (data not shown). Taken together, these results indicate that treating 4T1-bearing mice with FFLZ both concurrently with and after tumor cell injection inhibits breast cancer carcinogenesis.

### FFLZ inhibits the proliferation, colony formation and transformation capability of breast cancer cells *in vitro*

To evaluate the effects of FFLZ on the growth of mouse breast cancer cells, we next examined the effects of FFLZ on breast cancer cells *in vitro*. Treating 4T1 cells with FFLZ led to a decrease in viable cell number compared to the control (Fig. 1C). In addition, the effect of FFLZ on cell viability was examined by MTT assay; we found that 4T1 and MDA-MB-231 (a triple-negative human breast cancer line) cells exhibited significant sensitivity to FFLZ in a dose-dependent fashion (Fig. 1D, and Sup. Fig. 1D). Next, using soft agar assays, we found that FFLZ-treated 4T1 cells formed fewer colonies than untreated cells (Fig. 1E), indicating that FFLZ inhibits the colony formation of 4T1 cells. Together, the results indicate that FFLZ-treated breast cancer cells have less proliferation ability than control cells.

Because it has been reported that the activation of the Akt signaling pathway is involved in the regulation of survival mechanisms^21^, we tested the effect of FFLZ on the Akt pathway. The results of western blotting assays indicated that the endogenous phosphorylation of Akt in 4T1 and in MDA-MB-231 cells was down-regulated under 3-h FFLZ treatment (Fig. 1F).

### FFLZ inhibits spontaneous pulmonary metastasis in the tested mouse lungs and reduces the invasion of breast cancer cells

In cancer metastasis, multiple processes, including cell migration and invasion, are involved in the spread of primary tumors^22^. Because it is known that 4T1 cells exhibit higher spontaneous metastasis^23^, we were interested in testing whether FFLZ treatment of 4T1-bearing mice could suppress the metastatic ability of 4T1 cells. In these preliminary experiments, we found that on day 36 after the implantation of 4T1 cells into the BALB/c mice, metastatic lesions emerged on the examined organs and apparent tumor nodules occurred on the examined lungs (Sup. Fig. 2A,B).

**Figure 2:**
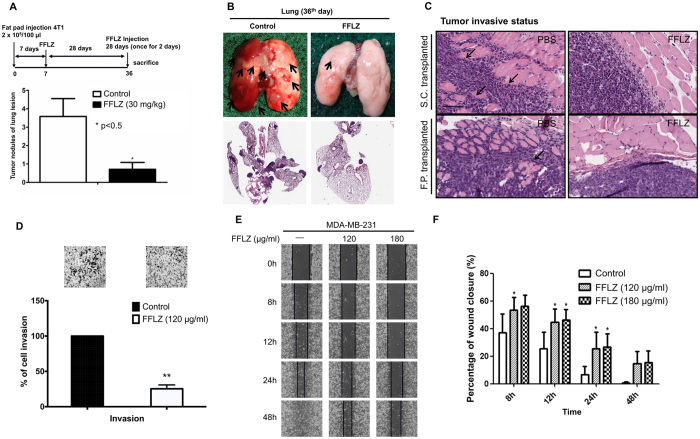
FFLZ inhibits spontaneous pulmonary metastasis in mouse lungs and reduces the invasion of breast cancer cells. (**A**) Balb/c mice were transplanted with 4T1 cells and treated with FFLZ (30 mg/kg) as described in Fig. 1. Tumor nodules of lung lesion in FFLZ post treatment experiment. (**B**) Pictures of lung lesions from 4T1-bearing mice and quantitation of the nodules of lung lesions. (**C**) Hematoxylin and eosin (H&E) staining from tumor lesions, smooth edge encircling indicated by black arrows. Scale bar, 100 μm. (**D**) Invasion ability of MDA-MB-231 cells was quantified by counting the number of cells that migrated to the underside of the porous polycarbonate membrane under microscopy and normalized to the control group. Data are expressed as the mean ± SD (N > 5) by Student’s t test; one of three independent experiments is presented. (**E**) Wound closure assays in MDA-MB-231 cells. Images were captured at 0–48 h in serum-free medium in the presence of FFLZ (0–180 μg/ml) or PBS. (**F**) Percentages of wound closure at 0–48 h under each condition as plotted, in which the wound width was normalized to the initial value at 0 h.

Later, extensive metastasis studies were conducted, and we found that fewer metastatic nodules emerged per lung in FFLZ-treated 4T1-bearing mice compared to control mice (Fig. 2A). Next, the results of H&E staining indicated that the number of pulmonary metastatic tumor lesions in the control mice increased significantly with extended time (Fig. 2B). Specifically, we found that tumors from 4T1-bearing mice formed 3.7 metastatic nodules per lung in the control mice; by contrast, approximately 0.9 metastatic nodules per lung formed in the FFLZ-treated mice (Fig. 2A). In addition, a smooth edge encircling the xenografted tumors appeared in the FFLZ-treated mice; in contrast, the encircled invasive status of xenografted tumors appeared in the PBS-treated mice in both the subcutaneous and fat pad experimental models (Fig. 2C). Moreover, the results of histological analyses showed similar results between spontaneous lung metastasis (Sup. Fig. 2A,B) and liver metastasis (Sup. Fig. 2C) in the tested 4T1-bearing mice.

To examine the migration properties of breast cancer cells, we performed wound closure assays and migration/invasion assays to determine the effect of FFLZ on cell mobility. Our results indicated that FFLZ significantly reduced human breast cancer MDA-MB-231 cell invasion (Fig. 2D) and mobility (Fig. 2E,F) *in vitro*, suggesting that such reduction is among one of the mechanisms by which FFLZ inhibits cell metastasis *in vivo*. Together, our results indicate that FFLZ remarkably reduces the number of metastatic nodules and micrometastasis in the lungs and liver, partly due to inhibiting breast cancer mobility and metastasis *in vivo* and *in vitro*.

### The decrease in TGFR-induced Smad-dependent pathway-relevant proteins is involved in the FFLZ-reduced expression of epithelial mesenchymal transition (EMT) markers

The reduction of cell-cell adhesion is another important event in cell invasion and metastasis^24^. We examined the effects of FFLZ on the expression of epithelial and mesenchymal markers and further investigated the mechanism of FFLZ-induced EMT morphological changes in breast cancer cells in terms of metastasis. In brief, MDA-MB-231 cells incubated with FFLZ showed a distinct cell-cell adhesion and cluster morphology compared to vehicle-treated MDA-MB-231 cells, which displayed a spindle-like morphology and revealed scattered dissemination (Sup. Fig. 3A). To determine the effects of FFLZ on actin cytoskeletal reorganization, MDA-MB-231 cells were treated with FFLZ for 24 h and stained for F-actin. We found that, compared with untreated cells, FFLZ induced morphological changes, including the formation of protrusions and the destruction of actin filaments (Sup. Fig. 3B).

**Figure 3:**
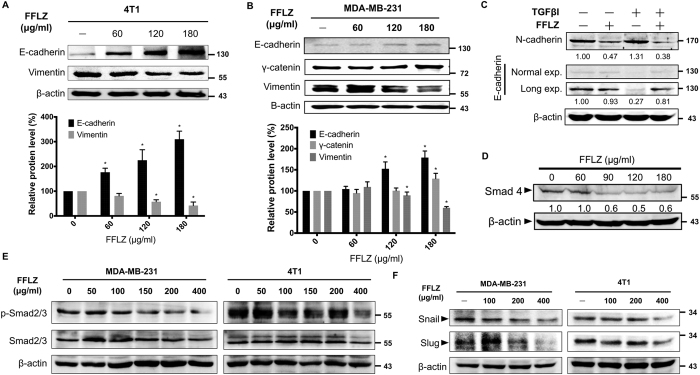
FFLZ reduces the expression of EMT markers via decreasing TGFR-induced Smad-dependent pathway-related proteins. (**A**) 4T1 and (**B**) MDA-MB-231. Total protein lysates derived from breast cancer cells treated with FFLZ for 48 h. Lysates were subjected to western blot analysis by antibodies against E-cadherin, vimentin, and γ-cadherin to determine protein expression of the indicated EMT markers. (**C**) Total protein lysates derived from MDA-MB-231 cells pre-treated with TGFβ for 24 h and then treated with FFLZ for 48 h. The indicated proteins were detected by western blot analysis. E-cadherin: Short and long exposure. (**D**) 4T1 cancer cells were incubated with FFLZ for 4 h, and whole cell lysates were analyzed by western blotting with anti-Smad4 antibody. (**E**) Breast cancer cells (1 × 10^5^ cells/ml) were incubated with FFLZ (0–400 μg/ml) for 4 h, and whole cell lysates were analyzed by western blotting with anti-phospho-Smad2 antibody. (**F**) Breast cancer cells (1 × 10^5^ cells/ml) were incubated with FFLZ (0–400 μg/ml) for 4 h, and whole cell lysates were analyzed by western blotting with anti-snail and anti-slug antibodies. All western blotting data represented one of three separate determinations in same experimental conditions.

In addition, during 24-h FFLZ treatment of cells, the up- and down-regulation of E-cadherin and Vimentin expression in 4T1 (Fig. 3A) and MDA-MB-231 cells (Fig. 3B) occurred in a dose-dependent manner. We extended the study of EMT marker expression in 4T1 cells and found that the enhanced expression of γ-catenin and the reduced expression of N-cadherin occurred within 24 h in a FFLZ dose-dependent manner (Sup. Fig. 3C).

Because TGFβ/TGFR signaling is involved in the EMT of cancer cells^24^, we found that FFLZ abolished the TGFβ1-induced alteration in EMT and rescued the cells to an epithelial phenotype by increasing E-cadherin, an important component of EMT, resulting in the inhibition of cell-cell adhesion in MDA-MB-231 cells (Fig. 3C). Moreover, the level of Smad4, which is a transcription factor for TGFβ/TGFR signaling also decreased upon FFLZ treatment (Fig. 3D). We found that FFLZ also reduced Smad2 phosphorylation, possibly through a reduction in TGFR proteins (Fig. 3E). We examined the expression levels of Snail and Slug, which are proteins that coordinate with the E-boxes in the E-cadherin proximal promoter to repress E-cadherin transcription^25^. The protein expression levels of Snail and Slug were down-regulated in FFLZ-treated MDA-MB-231 and 4T1 cells, most likely via the reduced phosphorylation of Smad2/3 (Fig. 3E,F).

Furthermore, we used TGFβ1 to trigger the downstream signaling of TGFβ pathway and found that FFLZ also down-regulates the TGFβ1-induced phosphorylation of Smad2/3 and the expression of Snail (Sup. Fig. 4A). Similar results were observed in treatment with TGFR inhibitor, SB431542 (Sup. Fig. 4B). These findings suggested that the phenomenon of morphology alteration was due to the ability of FFLZ to decrease mesenchymal cell markers (e.g., N-cadherin and vimentin) or increase epithelial cell markers (e.g., E-cadherin and γ-catenin). Moreover, FFLZ decreases the TGFβ-induced Smad2/3-Smad4-Snail/Slug-axis pathway and the expression of the EMT-related transcriptional factors in breast cancer cells.

### FFLZ accelerates ubiquitin-dependent proteasome-mediated degradation of TGFR (TGFRI and TGFRII) expression in FFLZ-treated MDA-MB-231 cells

**Figure 4:**
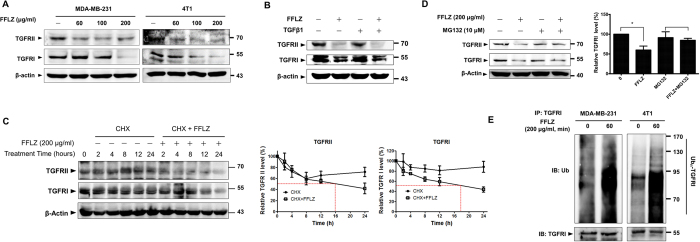
FFLZ accelerates ubiquitin-dependent proteasome-mediated degradation of TGFR (TGFRI and TGFRII) expression in FFLZ-treated MDA-MB-231 cells. (**A**) MDA-MB-231 and 4T1 cells were treated with FFLZ (60–200 μg/ml) for 1 h and harvested for immunoblotting analysis. (**B**) Total protein lysates derived from MDA-MB-231 cells treated with TGFβ for 24 h and FFLZ for 48 h, then analyzed by western blotting of TGFRI and II. (**C**) Time course of TGFRI and TGFRII degradation after addition of cycloheximide (CHX, 10 μg/ml) in the presence and absence of FFLZ (200 μg/ml) for 0 to 24 h in MDA-MB-231 cells (left panel). Quantification and normalization of TGFRs band intensities in an experiment representative of three separate determinations by ImageJ (right panel). Actin was used as an internal control. (**D**) Total protein lysates derived from MDA-MB-231 cells treated with MG132 for 0.5 h, and then with FFLZ for 1 h, then analyzed by western blotting of TGFRI and II. Quantification of bands in right panel (*p < 0.01, log-rank test for significance). (**E**) Detection of ubiquitin levels by immunoprecipitation of TGFRI, TGFRII in MDA-MB-231 cells pre-treated with MG-132 (10 μM) for 30 min, followed by additional treatment with FFLZ (200 μg/ml) for 60 min as indicated. After incubation, the whole cell lysates were immunoprecipitated (IP) overnight at 4 °C by anti-TGFRI antibody, followed by western blotting with anti-ubiquitin antibody. All western blotting data represented one of three separate determinations in same experimental conditions.

Previous studies have revealed that TGFβ/TGFRs mediate the activation of Smad- and/or non-Smad pathways, induce EMT and further promote tumor invasion and metastasis^26^. Here, we examined whether an alteration in TGFβ-Smad signaling is involved in FFLZ-mediated EMT. Prompted by this hypothesis, using western blotting analysis, we dissected the mechanism of the FFLZ-mediated down-regulation of TGFR (TGFRI and TGFRII) protein expression. Initially, we found that TGFR proteins dramatically decreased under the short-term FFLZ treatment of MDA-MB-231 and 4T1 cells (within 1 h, at 100 or 200 μg/ml), i.e., reduced amounts of TGFRI and II proteins were detected under FFLZ treatment compared to control cells (Fig. 4A). Similarly, we found that FFLZ reduced TGFR proteins in MDA-MB-231 cells during 48-h FFLZ treatment (Fig. 4B, samples 1 and 2); even in the presence of TGFβ1, we found that FFLZ also significantly reduced TGFR proteins in FFLZ-treated MDA-MB-231 cells (Fig. 4B, samples 3 and 4).

Second, to investigate the mechanism of the FFLZ-mediated degradation of TGFR proteins, we examined the (degradation) half-life (T½) of TGFRs in MDA-MB-231 cells treated with cycloheximide (CHX) to block *de novo* protein synthesis. We found a marked increase in TGFR turnover degradation rates in the presence of FFLZ in MDA-MB-231 and 4T1 cells (Fig. 4C and Sup. Fig. 5): the T½ values of TGFRI and TGFRII in MDA-MB-231cells treated with CHX+FFLZ were approximately 18 h and 16 h, respectively, shorter than the T½ values of TGFRI or TGFRII in cells treated with CHX alone (more than ~48 h) (Fig. 4C). Therefore, we proposed that FFLZ may enhance TGFRs degradation through modulation the stability of TGFRs. Next, by testing the effect of FFLZ treatment on TGFR proteins in the presence of the proteasome inhibitor MG132, we found that MG132 recovered the FFLZ-induced TGFR degradation (Fig. 4D, lane 4, FFLZ + MG132 *vs*. lane 2, FFLZ), i.e., more TGFRI and II proteins were found in FFLZ+MG132-treated cells (lane 4) than in FFLZ-treated cells (lane 2).

Third, an *in vitro* ubiquitination (ubiquitin) activity assay was used to examine the involvement of the ubiquitin-proteasome pathway (UPP) in the FFLZ-mediated proteasome degradation of TGFR proteins in MDA-MB-231 breast cancer cells. In brief, the cells were pre-incubated with MG132 and then treated with FFLZ, followed by the incubation of whole-cell lysates with anti-TGFR antibodies. The immunoprecipitated proteins were then analyzed via blotting with an anti-ubiquitin antibody. We found that degraded TGFRI protein could be detected with anti-ubiquitin antibodies in MDA-MB-231 and 4T1 cells (Fig. 4E). We demonstrated that the intensity of the smeared bands of degraded TGFRI protein in the FFLZ-treated cells was stronger than in control cells (Fig. 4E, samples 2 *vs*. 1, and 4 *vs*. 3), indicating that TGFRI in FFLZ-treated cells underwent a higher level of ubiquitination than in control cells during the FFLZ-induced decrease in TGFRI proteins. By contrast, there was less significant ubiquitination of TGFRII (data not shown). Taken together, these data are the first to demonstrate that UPP is involved in the FFLZ-mediated and -enhanced TGFR degradation in MDA-MB-231 cells.

### FFLZ modulates TGFRI protein in MDA-MB-231 cells by accelerating lipid rafts/caveolae-mediated endocytosis of TGFR

**Figure 5:**
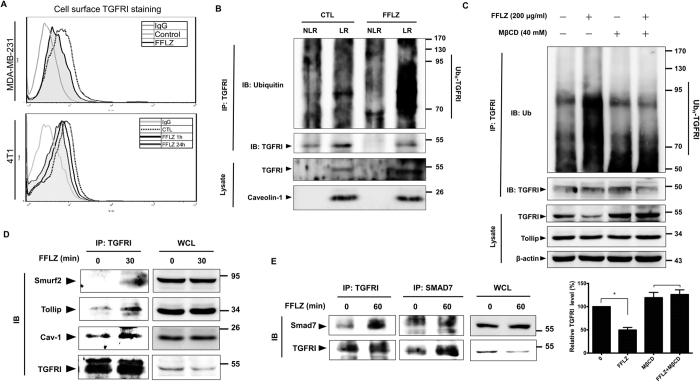
FFLZ modulates TGFRI protein in breast cancer cells via accelerating lipid rafts/caveolae-mediated endocytosis of TGFR. (**A**) MDA-MB-231 and 4T1 cells were treated with FFLZ (200 μg/ml) for 1–24 h, followed by analysis of TGFRI expression by FACS. (**B**) MDA-MB-231 cells were treated with FFLZ (60–200 μg/ml) for 1 h and lysed, and the lysates were subjected to sucrose gradient centrifugation for the isolation of lipid rafts fractions (LR) and non-lipid rafts fractions (NLR), followed by immunoprecipitation (IP) pull-down overnight and then western blotting with anti-ubiquitin antibody and TGFRI antibody. Caveolin-1 served as the marker for lipid rafts. (**C**) MDA-MB-231 cells were treated with MG132 for 0.5 h, followed by treatment with MβCD, a lipid rafts inhibitor, for 1 h and then treatment with FFLZ for 1 h. After incubation, the whole cell lysates (WCLs) were immunoprecipitated (IP) by anti-TGFRI antibody, followed by immunoblotting (IB) analysis with anti-ubiquitin antibody. Quantification of total lysates of TGFRI band intensities in experiment by ImageJ^®^, representing one of three separate determinations in same experimental conditions. (**D**) MDA-MB-231 cells were treated with FFLZ (200 μg/ml) for 30 min. After incubation, WCLs were harvested for IP as indicated and then analyzed by IB. (**E**) MDA-MB-231 cells were treated with FFLZ (200 μg/ml) for 60 min. After incubation, WCLs were harvested for IP and IB analysis, with WCL as loading control. All western blotting data represented one of three separate determinations in same experimental conditions.

Using FACS to detect TGFR protein expression on the surface of the cell membrane, we confirmed that FFLZ reduced the expression of TGFRs on the membranes of both MDA-MB-231 and 4T1 cells in a short time (Fig. 5A). In brief, the TGFRs protein level on the cell membrane was significantly decreased in FFLZ-treated cells compared to control cells within 1 h.

Because it has been reported that TGFRI ubiquitination is also promoted via lipid rafts/caveolae-mediated endocytosis^27^, we further examined whether lipid rafts/caveolae were involved in the FFLZ-mediated degradation of TGFRI. Using sucrose gradient centrifugation to isolate lipid rafts (LR) and non-lipid rafts fractions (NLR) from MDA-MB-231 cells, we found that FFLZ induced TGFR co-localization to lipid rafts fractions (Fig. 5B, lane 2 *vs*. 4, total lysate). We also found that TGFRI protein was dramatically increased in the LR fraction of FFLZ-treated cells, and much more ubiquitinated TGFRI was immunoprecipitated from the FFLZ-treated lipid rafts/caveolin fractions than from the control groups (Fig. 5B, lane 2 *vs*. 4, IP: TGFRI). Compared with normal conditions and the results of FACS (Fig. 5A), we found that FFLZ promoted the TGFRI protein on the membrane to “enter” the lipid rafts/caveolin fraction and further facilitated endocytosis and the UPP-mediated degradation pathway (Fig. 5B). Based on the results of lipid-raft involvement in TGFRI degradation, we used methyl-β-cyclodextrin (MβCD) to inhibit lipid rafts formation and found that, as expected, the level of TGFRI protein in FFLZ-treated cells was rescued, i.e., more TGFRI protein was present after FFLZ+MβCD treatment than after FFLZ treatment alone (Fig. 5C, lanes 4 *vs*. 2, total lysate). Furthermore, the intensity of the smeared bands of degraded TGFRI protein in the FFLZ-treated cells was stronger than in the MβCD-treated cells (Fig. 5C, lane 4 *vs*. 2, IP: TGFRI). Taken together, these data are the first to demonstrate that lipid rafts/caveolae-mediated endocytosis is involved in FFLZ-modulated ubiquitin-proteasome pathways and ubiquitin-enhanced TGFRI degradation.

### FFLZ promotes caveolin-dependent degradation by triggering the Smurf2/Smad7/Tollip complex to conjugate to TGFRI

The conjugation of Smurf2 and Smad7 to TGFRI performs critical functions in modulating the intracellular trafficking and degradation of ubiquitinated TGFRI but negatively regulates TGFβ signaling pathways^28^. By examining the mechanism of Smurf2 involvement in FFLZ-induced TGFRI degradation, we found that FFLZ enhanced Smurf2 and caveolin-1 binding to TGFRI, which regulates the lipid rafts/caveolae-mediated endocytosis pathway (Fig. 5D). In addition, we found that FFLZ induced Smad7 conjugation to TGFRI (Fig. 5E). These results indicate that FFLZ induces the Smurf2/Smad7/TGFR-complex to process the UPP degradation pathway of TGFRI.

Toll-interacting protein (Tollip), an inhibitory adaptor protein within the toll-like receptor (TLR) pathway, cooperates with Smad7 and Smurf2 to modulate intracellular trafficking and promote the degradation of ubiquitinated TGFRI by stabilizing the ubiquitylated chain of TGFR, negatively regulating TGFβ signaling pathways^29^. Interestingly, using a co-immunoprecipitation assay of Tollip and TGFRI, we found that FFLZ enhanced the interaction between TGFRI and Tollip (Fig. 5D). To further examine the role of Tollip in FFLZ-induced TGFRI degradation, the expression of Tollip in MDA-MB-231 cells was knocked down via shRNA (Sup. Fig. 6A). We found that the TGFRI protein was recovered in Tollip-shRNA groups (Sup. Fig. 6A, mock *vs*. samples #24 and #41). Moreover, we also found that transfection of Tollip-shRNA effectively blocked FFLZ-induced TGFRI degradation (Sup. Fig. 6B, sample 2 *vs*. 4). Together, our findings indicate that the specific Smurf2/Smad7/Tollip axis is involved in the FFLZ-induced ubiquitination-mediated degradation of TGFRI, as shown in proposed scheme (Sup. Fig. 7).

### Study of the synergistic effects of combining FFLZ and trastuzumab (Herceptin^®^) in the inhibition of BT474 and SKBR3 trastuzumab-resistant human breast cancer cells

**Figure 6:**
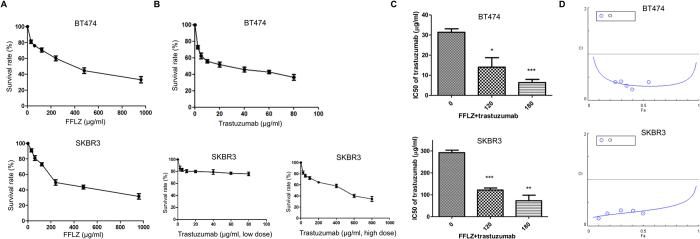
Synergistic anti-cancer activity of the combination of FFLZ and trastuzumab in BT474 and SKBR1 human breast cancer cells. (**A)** Human breast cancer (BT474 and SKBR3) cells with a density of 2 × 10^5^ cells/dish were treated with various concentrations of FFLZ (30–960 μg/ml) for 72 h, and the viability of cells was determined by MTT assay to find the IC_50_ of breast cancer cells. (**B**) Human breast cancer cells (BT474 and SKBR3) were treated with trastuzumab (2.5–800 μg/ml) for 72 h. Trastuzumab-resistant SKBR3 cells were treated with low- and high-dose trastuzumab. (**C**) The IC_50_ of breast cancer cells was determined by co-treating with trastuzumab and various dosages of FFLZ, and the viability of cells was determined by the MTT assay. Each bar represents the mean ± SD of three experiments. *P < 0.05, ***P < 0.005 compared to the control group. (**D**) CI values were evaluated by the “CompuSyn” software and expressed as in the figures.

In addition, a significant number of patients with triple-negative cancer (similar to the MDA-MB-231 cell line) or trastuzumab-resistant cancer (similar to the trastuzumab-resistant SKBR3 cell line) do not benefit from targeted therapy with trastuzumab^20^. Here, the results suggested for FFLZ and trastuzumab in inhibiting the cell viability of other types of breast cancer cells, SKBR3 (HER2-positive, trastuzumab-resistant) and BT474 (triple positive) in a dose-dependent manner (Fig. 6A,B). Moreover, the interaction between FFLZ and trastuzumab was further evaluated using the CompuSyn^TM^ program combination index (CI) developed by Chou-Talalay; in brief, CI < 1 indicates synergy, CI = 1 indicates additivity, and CI > 1 indicates antagonism^30^. This algorithm estimates the CI value for each dose based on the results expected from each of the single agents. Specifically, after 72-h treatment of cells with the combination of FFLZ and trastuzumab, we found that the IC_50_ was decreased dramatically by the combination treatment of FFLZ and trastuzumab in BT474 and SKBR3 (Fig. 6C). The CI values, i.e., CI < 0.4 (BT474) and 0.3 (SKBR3), indicated that the combination of FFLZ and trastuzumab synergistically decreased tumor cell viability *in vitro* (Fig. 6D).

## Discussion

*Ganoderma lucidum*, which contains polysaccharides including FFLZ, is applied as a dietary supplement with chemotherapeutic drugs for anticancer activity^31^,^32^,^33^,^34^; however, the mechanism of FFLZ in anti-breast cancer has not been elucidated. According to our results, the TGFRI protein level is dramatically decreased in a short time upon FFLZ treatment, similarly to our previous study of fucoidan (a polysaccharide from brown seaweeds)^35^. We proposed that FFLZ facilitates the degradation of TGFRs through the proteasome or lysosome; however, ubiquitination processing may not always be involved in the lysosomal degradation of TGFR^36^. Using MG132, we confirmed that the proteasome pathway participates in FFLZ-mediated TGFRI degradation. Moreover, our current results indicate that FFLZ triggers ubiquitin-dependent proteasomal pathways (UPP) in the down-regulation of TGFR expression. As is well known, Smurf2 is among the most important E3 ligases in regulating TGFR degradation^16^. Based on our current findings, we are one of the first to report that FFLZ triggers Smurf2 and Smad7 binding and recruitment to TGFRI, enhancing the UPP of TGFR, which is consistent with our previous reports on fucoidan^16^,^37^.

Toll-interacting protein (Tollip) is a regulator of the Toll-like receptor (TLR)-mediated signaling pathway, including pro-inflammatory responses. Tollip can associate with TLRs and modulate TLR-mediated cell activation^38^. It was recently reported that Tollip negatively regulates TGFR signaling pathways by stabilizing the ubiquitin-chain of TGFRI^29^. Therefore, we proposed that FFLZ may enhance TGFRs degradation through modulation the stability of TGFRs. Interestingly, our findings also suggest that FFLZ enhances the cooperation of Tollip with the TGFRI/Smurf2/Smad7 complex and further modulates the intracellular trafficking and degradation of ubiquitinated TGFRI, thus negatively regulating TGFβ signaling pathways. Recent reports have also indicated that the overexpression of TLR4 in human breast cancer often correlates with chemoresistance and metastasis^39^,^40^,^41^. Moreover, we have previously reported that FFLZ enhances the CD14 endocytosis of LPS and promotes TLR4 signal transduction^42^. Therefore, we proposed that the TLR4/Tollip axis may involve fucose-containing polysaccharides-induced UPP in the down-regulation of TGFR expression and related signaling pathways in the breast cancer cells, leading to decreased cancer cell proliferation and metastasis.

In general, the lipid rafts/caveolae-mediated and non-lipid rafts/clathrin-mediated pathways participate in the regulation of TGFR functions^43^. We previously reported that polysaccharides can reduce lung cancer tumorigenesis^2^, and fucose-rich polysaccharides extracted from brown seaweeds (fucoidan) potentially suppress breast cancer metastasis^44^. Here, we first found that FFLZ enhances the translocation of TGFRI to the lipid rafts/caveolae fraction via sucrose gradient centrifugation. Specifically, we demonstrated that FFLZ increases the inhibition of TGFβ signaling via promoting TGFR trafficking to the caveolin-1-rich lipid-raft fraction. Interestingly, both structure of FFLZ and fucoidan contain fucose residues in side chains or backbone of polysaccharide. Therefore, these data implied that polysaccharides containing fucose residues may modulate the degradation and translocation of TGFRs. Furthermore, our results indicated that FFLZ disrupts the balance of lipid-raft fractions and promotes the localization of the TGFRs to the caveolae, which facilitates UPP-mediated TGFR degradation.

In addition, it has been shown that the clathrin-coated early endosome promotes TGFβ-induced Smad and non-Smad (e.g., phosphatidylinositol-3-kinase/AKT pathways) activation and transcriptional responses^43^. Metastatic breast cancer cells also tend to enter the clathrin-mediated (EEA-1-conjugated) early endosome to modulate the TGFR signaling pathways^45^. As is already known, in TGFβ-mediated non-Smad signal transductions, TGFβ induces the Akt/mTOR pathway to promote cancer cell proliferation and invasion^46^. In our current study, we found that FFLZ inhibits Akt and PI3K phosphorylation, indicating that FFLZ directly down-regulates the TGFR non-Smad pathway and consequently inhibits survival signaling in breast cancer cells. In addition, FFLZ inhibits the invasion of breast cancer cells during the EMT through suppressing the Smad2/3 signal transductions. Thus, we found that FFLZ enhances the UPP of TGFRI but reduces TGFR-mediated Smad and non-Smad signal transduction.

On the other hand, as FFLZ shows lower toxicity in normal cells (data not shown), we tentatively examined the potential of using FFLZ as a dietary supplement for breast cancer patients. We intended to combine FFLZ and trastuzumab to target trastuzumab-resistant patients. Initially, we tested whether FFLZ and/or trastuzumab inhibits breast cancer cells, BT474, and trastuzumab-resistant cells, SKBR3. The inhibitory effects of FFLZ and trastuzumab on breast cancer cells covered a broad range (Fig. 6). In addition, we observed sensitization of breast cancer cells under the combination of trastuzumab and FFLZ. Importantly, the IC50 values of trastuzumab were significantly reduced by co-treatment with various concentrations of FFLZ, suggesting that the cytotoxicity of trastuzumab to breast cancer cells was synergistically enhanced under the combined usage of trastuzumab and FFLZ. Mechanistically, we propose that this combination could use trastuzumab and FFLZ as a HER2 inhibitor and inducer of TGFR degradation, respectively. In conclusion, we have clarified the mechanisms of the FFLZ-induced lipid rafts/Smurf2-dependent ubiquitin degradation of TGFRs and hope that FFLZ might be a potential therapeutic or dietary supplemental agent. Furthermore, we confirmed the synergistic effect of trastuzumab and FFLZ, and we hope for another potential trial for their clinical application and benefits to human breast cancer patients.

## Methods

### Cell cultures

MDA-MB-231 human breast cancer cells and BALB/c mouse-derived 4T1 mammary adenocarcinoma cells (4T1) were obtained from the Cell Bank of the Bioresource Collection and Research Center (Taiwan). SKBR3 and BT474 human breast cancer cells were obtained from Dr. L.-M. Tseng (Taipei Veterans General Hospital, Taiwan). The cell lines were maintained in Dulbecco’s modified Eagle’s medium (DMEM) with 10% heat-inactivated fetal bovine serum, 200 mM L-glutamine and 1× non-essential amino acids. The cells were maintained in an incubator with a 5% CO_2_ atmosphere at 37 °C.

### Reagents and antibodies

Recombinant human TGFβ1 was purchased from R&D Systems (USA). The proteasome inhibitor MG132 was purchased from Merck Millipore (USA). Cycloheximide (CHX), Methyl-β-cyclodextrin (MβCD) and TGFRI inhibitor (SB431542) was purchased from Sigma-Aldrich (USA). Trastuzumab, an anti-HER2 monoclonal antibody, was purchased from Roche (Switzerland). Antibodies against p-Akt, Akt, p-Smad2/3, Smad2/3, Smad4, β-actin, TGFRI (immunoprecipitation), TGFRII, and γ-catenin, as well as normal rabbit IgG, anti-rabbit, anti-mouse, and anti-rat IgG-HRP antibodies and protein A/G plus agarose, were obtained from Santa Cruz Biotechnology (USA). Anti-rabbit IgG-DyLight 488 was obtained from Thermo Fisher Scientific (USA). Antibodies against Caveolin-1, E-cadherin and N-cadherin were obtained from BD Transduction Laboratories (USA). An antibody against Vimentin was purchased from Thermo Scientific (USA). An antibody against Snail was purchased from Abcam Plc. (UK). An antibody against Slug was purchased from Cell Signaling Technology, Inc. (USA). An antibody against Smurf2, Smad7, Tollip, ubiquitin and TGFRI (immunoblotting) was purchased from GeneTex (USA).

### Preparation of the fucose-containing fraction of Ling-Zhi (FFLZ)

*G. lucidum* raw material (Ling-Zhi) was obtained from Wyntek Corporation (Taiwan). Two additional steps were required for the purification of the Ling-Zhi extract, namely the preparation of a crude extract and the preparation of the final Ling-Zhi extract. The compounds in the crude extract and in the Ling-Zhi extract were monitored by high-pressure liquid chromatography using a size exclusion column Tosoh TSKgel G5000PWXL. Detailed information about FFLZ and the preparations of the *G. lucidum* polysaccharide extract has been previously reported^1^,^2^.

### Cell extracts and western blotting

The breast cancer cells or other indicated cells were grown on 6-cm tissue culture plates and were treated with FFLZ for the indicated times. The cells were then washed once with 1 ml of phosphate-buffered saline (PBS) containing 1% Na_3_VO_4_ and were lysed using 40 μl of lysis buffer. The detailed procedure was as previously described^35^. For the isolation and immunoprecipitation of proteins in lipid rafts/caveolae-mediated endocytic vesicles, cells were subjected to sucrose gradient centrifugation as previously described^47^.

### Cell viability assay (MTT assay)

Cancer cells (1 × 10^4^ cells per well) were seeded in triplicate on a 96-well plate and incubated overnight before treatment with FFLZ for 24–48 h. After incubation, 3-(4,5-dimethylthiazol-2-yl)-2,5-diphenyltetrazolium bromide (MTT) dye was added, and the mixture was incubated for 2 h as previously described^44^.

### Colony formation

Breast cancer cells were seeded at 3000 cells per well in agar solution in six-well plates for 21–28 days. After incubation, the cells were dyed by Giemsa staining at room temperature. After the dye was washed off, the plates were photographed. The detailed procedure was as previously described^35^.

### Cell migration and invasion

Cell migration assays were performed using 6.5-mm Costar Transwell^®^ chambers (8-μm pore size; Corning, USA). After treatment with FFLZ (120 μg/ml) for 24 h followed by stimulation with exogenous TGFβ1 (5 ng/ml) for 24 h, the cells (4 × 10^4 ^cells/200 μl) were seeded into Transwell^®^ chambers. The detailed procedure was as previously described^35^.

### Breast cancer animal model

BALB/c mice (6–8 weeks of age) were obtained from the National Laboratory Animal Center (Taiwan). Each mouse received an injection of 2 × 10^5^ 4T1 cells into the mammary fat pads. To examine the effects of FFLZ on tumor volume, the mice were randomly distributed into two groups: PBS-treated (control) and FFLZ-treated (30 mg/kg). The treatment period was approximately 5 weeks (days 0–36), and we recorded the body weight and tumor size (calculated as length × width × height in mm^3^) of each mouse every 2 days. At the end of the treatment, the livers, lungs, and tumors were collected after the mice were sacrificed. Lungs and livers were fixed in 10% formalin, and we counted the visible lung surface macrometastatic lesions, which appeared as white spots.

Mice were raised under pathogen-free conditions in the Animal Center of National Yang-Ming University (NYMU). All experimental methods involving animals were carried out in accordance with guidelines and regulations of the Institutional Animal Care and Use Committee (IACUC) of NYMU, and all experimental protocols were approved by the IACUC of NYMU.

### Immunofluorescence analysis

The expression of TGFRs on the cell membrane was determined using flow cytometry. Cells were analyzed by double immunofluorescence using antibodies against human TGFRI (GeneTex, USA) and TGFRII (Cell Signaling, USA). The detailed procedure was as previously described^27^.

### Synergy analysis

Following drug treatment *in vitro*, the number of viable cells was measured using the MTT assay as described above. The data were normalized to the control group and expressed as the percentage of viability. Next, the collected data were analyzed using the CompuSyn^TM^ program (Biosoft, USA) based on the median-effect principle of Chou and Talalay^48^. The program calculated a combination index (CI), which was used to identify the tested drug interaction as synergistic, additive, or antagonistic.

### Design and transfection of specific short hairpin RNA oligonucleotides (shRNAs) targeting Tollip

Based on the sequence of the human Tollip gene (NM_019009), three specific shRNAs targeting Tollip were obtained from the National RNAi Core Facility Platform (Academia Sinica, Taiwan). To produce stable clones of Tollip-knockdown cell lines, we used the *in vitro* gene delivery of lentiviral vectors. The targeting sequences for Tollip are shown in Supplementary Table S1. The detailed procedure was as previously described^35^.

### Statistical analysis

All the data are expressed as the means ± SEM or SD. Significant differences between two groups were determined by t-test analyses. A *P* value of <0.05 was considered statistically significant.

## Additional Information

**How to cite this article**: Tsao, S.-M. and Hsu, H.-Y. Fucose-containing fraction of Ling-Zhi enhances lipid rafts-dependent ubiquitination of TGFβ receptor degradation and attenuates breast cancer tumorigenesis. *Sci. Rep*. **6**, 36563; doi: 10.1038/srep36563 (2016).

**Publisher’s note:** Springer Nature remains neutral with regard to jurisdictional claims in published maps and institutional affiliations.

## Supplementary Material

Supplementary Information

## References

[b1] Hsu H. Y. . Extract of Reishi polysaccharides induces cytokine expression via TLR4-modulated protein kinase signaling pathways. Journal of immunology 173, 5989–5999 (2004). 10.4049/jimmunol.173.10.5989 15528333

[b2] Liao S. F. . Immunization of fucose-containing polysaccharides from Reishi mushroom induces antibodies to tumor-associated Globo H-series epitopes. Proceedings of the National Academy of Sciences of the United States of America 110, 13809–13814, 10.1073/pnas.1312457110 (2013). 23908400 PMC3752246

[b3] Ma L. Role of miR-10b in breast cancer metastasis. Breast Cancer Res 12, 210, 10.1186/bcr2720 (2010). 21067538 PMC3096969

[b4] Bendre M., Gaddy D., Nicholas R. W. & Suva L. J. Breast cancer metastasis to bone: it is not all about PTHrP. Clinical orthopaedics and related research , S39–S45, 10.1097/01.blo.0000093844.72468.f4 (2003). 14600591

[b5] Bhola N. E. . TGF-beta inhibition enhances chemotherapy action against triple-negative breast cancer. The Journal of clinical investigation 123, 1348–1358, 10.1172/JCI65416 (2013). 23391723 PMC3582135

[b6] Buck M. B. & Knabbe C. TGF-beta signaling in breast cancer. Annals of the New York Academy of Sciences 1089, 119–126, 10.1196/annals.1386.024 (2006). 17261761

[b7] Moses H. & Barcellos-Hoff M. H. TGF-beta biology in mammary development and breast cancer. Cold Spring Harbor perspectives in biology 3, a003277, 10.1101/cshperspect.a003277 (2011). 20810549 PMC3003461

[b8] Scollen S. . TGF-beta signaling pathway and breast cancer susceptibility. Cancer epidemiology, biomarkers & prevention: a publication of the American Association for Cancer Research, cosponsored by the American Society of Preventive Oncology 20, 1112–1119, 10.1158/1055-9965.EPI-11-0062 (2011). PMC3112459 21527583

[b9] Siegel P. M. & Massague J. Cytostatic and apoptotic actions of TGF-beta in homeostasis and cancer. Nat Rev Cancer 3, 807–821, 10.1038/nrc1208 (2003). 14557817

[b10] Galliher A. J., Neil J. R. & Schiemann W. P. Role of transforming growth factor-beta in cancer progression. Future Oncol 2, 743–763, 10.2217/14796694.2.6.743 (2006). 17155901

[b11] Wakefield L. M. & Roberts A. B. TGF-beta signaling: positive and negative effects on tumorigenesis. Curr Opin Genet Dev 12, 22–29 (2002). 11790550 10.1016/s0959-437x(01)00259-3

[b12] Zhang Y. E. Non-Smad pathways in TGF-beta signaling. Cell research 19, 128–139, 10.1038/cr.2008.328 (2009). 19114990 PMC2635127

[b13] Thiery J. P. & Sleeman J. P. Complex networks orchestrate epithelial-mesenchymal transitions. Nature reviews. Molecular cell biology 7, 131–142, 10.1038/nrm1835 (2006). 16493418

[b14] Gupta G. P. & Massague J. Cancer metastasis: building a framework. Cell 127, 679–695, 10.1016/j.cell.2006.11.001 (2006). 17110329

[b15] Izzi L. & Attisano L. Regulation of the TGFbeta signalling pathway by ubiquitin-mediated degradation. Oncogene 23, 2071–2078, 10.1038/sj.onc.1207412 (2004). 15021894

[b16] Kavsak P. . Smad7 binds to Smurf2 to form an E3 ubiquitin ligase that targets the TGF beta receptor for degradation. Molecular cell 6, 1365–1375 (2000). 11163210 10.1016/s1097-2765(00)00134-9

[b17] Kang J. S., Liu C. & Derynck R. New regulatory mechanisms of TGF-beta receptor function. Trends in cell biology 19, 385–394, 10.1016/j.tcb.2009.05.008 (2009). 19648010

[b18] Le Roy C. & Wrana J. L. Clathrin- and non-clathrin-mediated endocytic regulation of cell signalling. Nature reviews. Molecular cell biology 6, 112–126, 10.1038/nrm1571 (2005). 15687999

[b19] Wang S. E. The Functional Crosstalk between HER2 Tyrosine Kinase and TGF-beta Signaling in Breast Cancer Malignancy. Journal of signal transduction 2011, 804236, 10.1155/2011/804236 (2011). 21637380 PMC3101605

[b20] Pohlmann P. R., Mayer I. A. & Mernaugh R. Resistance to Trastuzumab in Breast Cancer. Clinical cancer research: an official journal of the American Association for Cancer Research 15, 7479–7491, 10.1158/1078-0432.CCR-09-0636 (2009). 20008848 PMC3471537

[b21] Kretzschmar M., Doody J., Timokhina I. & Massague J. A mechanism of repression of TGFbeta/Smad signaling by oncogenic Ras. Genes & development 13, 804–816 (1999). 10197981 10.1101/gad.13.7.804 PMC316599

[b22] Hanahan D. & Weinberg R. A. Hallmarks of cancer: the next generation. Cell 144, 646–674, 10.1016/j.cell.2011.02.013 (2011). 21376230

[b23] Pulaski B. A. & Ostrand-Rosenberg S. Mouse 4T1 breast tumor model. Current protocols in immunology edited by [Coligan John E. . ] Chapter 20, Unit 20 22, 10.1002/0471142735.im2002s39 (2001). 18432775

[b24] Xu J., Lamouille S. & Derynck R. TGF-beta-induced epithelial to mesenchymal transition. Cell research 19, 156–172, 10.1038/cr.2009.5 (2009). 19153598 PMC4720263

[b25] Batlle E. . The transcription factor snail is a repressor of E-cadherin gene expression in epithelial tumour cells. Nature cell biology 2, 84–89, 10.1038/35000034 (2000). 10655587

[b26] Le Scolan E. . Transforming growth factor-beta suppresses the ability of Ski to inhibit tumor metastasis by inducing its degradation. Cancer research 68, 3277–3285, 10.1158/0008-5472.CAN-07-6793 (2008). 18451154

[b27] Zuo W. . c-Cbl-mediated neddylation antagonizes ubiquitination and degradation of the TGF-beta type II receptor. Molecular cell 49, 499–510, 10.1016/j.molcel.2012.12.002 (2013). 23290524

[b28] Itoh S. & ten Dijke P. Negative regulation of TGF-beta receptor/Smad signal transduction. Current opinion in cell biology 19, 176–184, 10.1016/j.ceb.2007.02.015 (2007). 17317136

[b29] Zhu L. . Tollip, an intracellular trafficking protein, is a novel modulator of the transforming growth factor-beta signaling pathway. The Journal of biological chemistry 287, 39653–39663, 10.1074/jbc.M112.388009 (2012). 23027871 PMC3501082

[b30] Chou T. C. Theoretical basis, experimental design, and computerized simulation of synergism and antagonism in drug combination studies. Pharmacological reviews 58, 621–681, 10.1124/pr.58.3.10 (2006). 16968952

[b31] Chang R. The central importance of the beta-glucan receptor as the basis of immunologic bioactivity of Ganoderma polysaccharides . (Seoul: II Yang Press, 1996).

[b32] Czop J. K. & Austen K. F. A beta-glucan inhibitable receptor on human monocytes: its identity with the phagocytic receptor for particulate activators of the alternative complement pathway. J Immunol 134, 2588–2593 (1985). 2579146

[b33] Chen H. S. . Studies on the immuno-modulating and anti-tumor activities of Ganoderma lucidum (Reishi) polysaccharides. Bioorganic & medicinal chemistry 12, 5595–5601, 10.1016/j.bmc.2004.08.003 (2004). 15465337

[b34] Yuen J. W. & Gohel M. D. Anticancer effects of Ganoderma lucidum: a review of scientific evidence. Nutrition and cancer 53, 11–17, 10.1207/s15327914nc5301_2 (2005). 16351502

[b35] Hsu H. Y. . Fucoidan inhibition of lung cancer *in vivo* and *in vitro*: role of the Smurf2-dependent ubiquitin proteasome pathway in TGFbeta receptor degradation. Oncotarget 5, 7870–7885 (2014). 25149540 10.18632/oncotarget.2317 PMC4202167

[b36] Huang F. & Chen Y. G. Regulation of TGF-beta receptor activity. Cell & bioscience 2, 9, 10.1186/2045-3701-2-9 (2012). 22420375 PMC3333473

[b37] Ogunjimi A. A. . Regulation of Smurf2 ubiquitin ligase activity by anchoring the E2 to the HECT domain. Molecular cell 19, 297–308, 10.1016/j.molcel.2005.06.028 (2005). 16061177

[b38] Zhang G. & Ghosh S. Negative regulation of toll-like receptor-mediated signaling by Tollip. The Journal of biological chemistry 277, 7059–7065, 10.1074/jbc.M109537200 (2002). 11751856

[b39] Yang C. X., Li C. Y. & Feng W. Toll-like receptor 4 genetic variants and prognosis of breast cancer. Tissue antigens 81, 221–226, 10.1111/tan.12096 (2013). 23510418

[b40] Ahmed A., Redmond H. P. & Wang J. H. Links between Toll-like receptor 4 and breast cancer. Oncoimmunology 2, e22945, 10.4161/onci.22945 (2013). 23526132 PMC3601164

[b41] Rajput S., Volk-Draper L. D. & Ran S. TLR4 is a novel determinant of the response to paclitaxel in breast cancer. Molecular cancer therapeutics 12, 1676–1687, 10.1158/1535-7163.mct-12-1019 (2013). 23720768 PMC3742631

[b42] Hua K.-F. . Ganoderma lucidum polysaccharides enhance CD14 endocytosis of LPS and promote TLR4 signal transduction of cytokine expression. Journal of Cellular Physiology 212, 537–550, 10.1002/jcp.21050 (2007). 17474083

[b43] Chen Y. G. Endocytic regulation of TGF-beta signaling. Cell research 19, 58–70, 10.1038/cr.2008.315 (2009). 19050695

[b44] Hsu H. Y. . Fucoidan induces changes in the epithelial to mesenchymal transition and decreases metastasis by enhancing ubiquitin-dependent TGFbeta receptor degradation in breast cancer. Carcinogenesis 34, 874–884, 10.1093/carcin/bgs396 (2013). 23275155

[b45] Zavadil J. & Bottinger E. P. TGF-beta and epithelial-to-mesenchymal transitions. Oncogene 24, 5764–5774, 10.1038/sj.onc.1208927 (2005). 16123809

[b46] Lamouille S. & Derynck R. Cell size and invasion in TGF-beta-induced epithelial to mesenchymal transition is regulated by activation of the mTOR pathway. The Journal of cell biology 178, 437–451, 10.1083/jcb.200611146 (2007). 17646396 PMC2064840

[b47] Zuo W. & Chen Y. G. Specific activation of mitogen-activated protein kinase by transforming growth factor-beta receptors in lipid rafts is required for epithelial cell plasticity. Molecular biology of the cell 20, 1020–1029, 10.1091/mbc.E08-09-0898 (2009). 19056678 PMC2633387

[b48] Chou T. C. & Talalay P. Quantitative analysis of dose-effect relationships: the combined effects of multiple drugs or enzyme inhibitors. Advances in enzyme regulation 22, 27–55 (1984). 6382953 10.1016/0065-2571(84)90007-4

