# Supplementary material for: Fucose-containing fraction of Ling-Zhi enhances lipid rafts-dependent ubiquitination of TGFβ receptor degradation and attenuates breast cancer tumorigenesis
Source: Sci Rep. 2016 Nov 10;6:36563. doi: 10.1038/srep36563 (PMC5103195; doi:10.1038/srep36563)
Supplement: Supplementary Information [file srep36563-s1.doc]

**Manuscript submission tracking number: SREP-16-18731**

**Title: Fucose-containing fraction of Ling-Zhi enhances lipid rafts- dependent ubiquitination of TGFβ receptor degradation and attenuates breast cancer tumorigenesis**

Order of Authors: Shu-Ming Tsao; Hsien-Yeh Hsu*

**Supplementary information**

## Supplementary Table I. Sequences of shRNA

| shRNA | Target sequence (5’ to 3’) | shRNA sequence (5’ to 3’) |
| --- | --- | --- |
| Tollip #17 (shTollip) | CCAACAAGATTCCCGTGAAAG | CCGGCCAACAAGATTCCCGTGAAAGCTCGAGCTTTCACGGGAATCTTGTTGGTTTTTG |
| Tollip #24 (shTollip) | TCGAGATCTTCGATGAGAGAG | CCGGTCGAGATCTTCGATGAGAGAGCTCGAGCTCTCTCATCGAAGATCTCGATTTTTG |
| Tollip #41 (shTollip) | GAACAAGGATGCCGCCATCAA | CCGGGAACAAGGATGCCGCCATCAACTCGAGTTGATGGCGGCATCCTTGTTCTTTTTG |


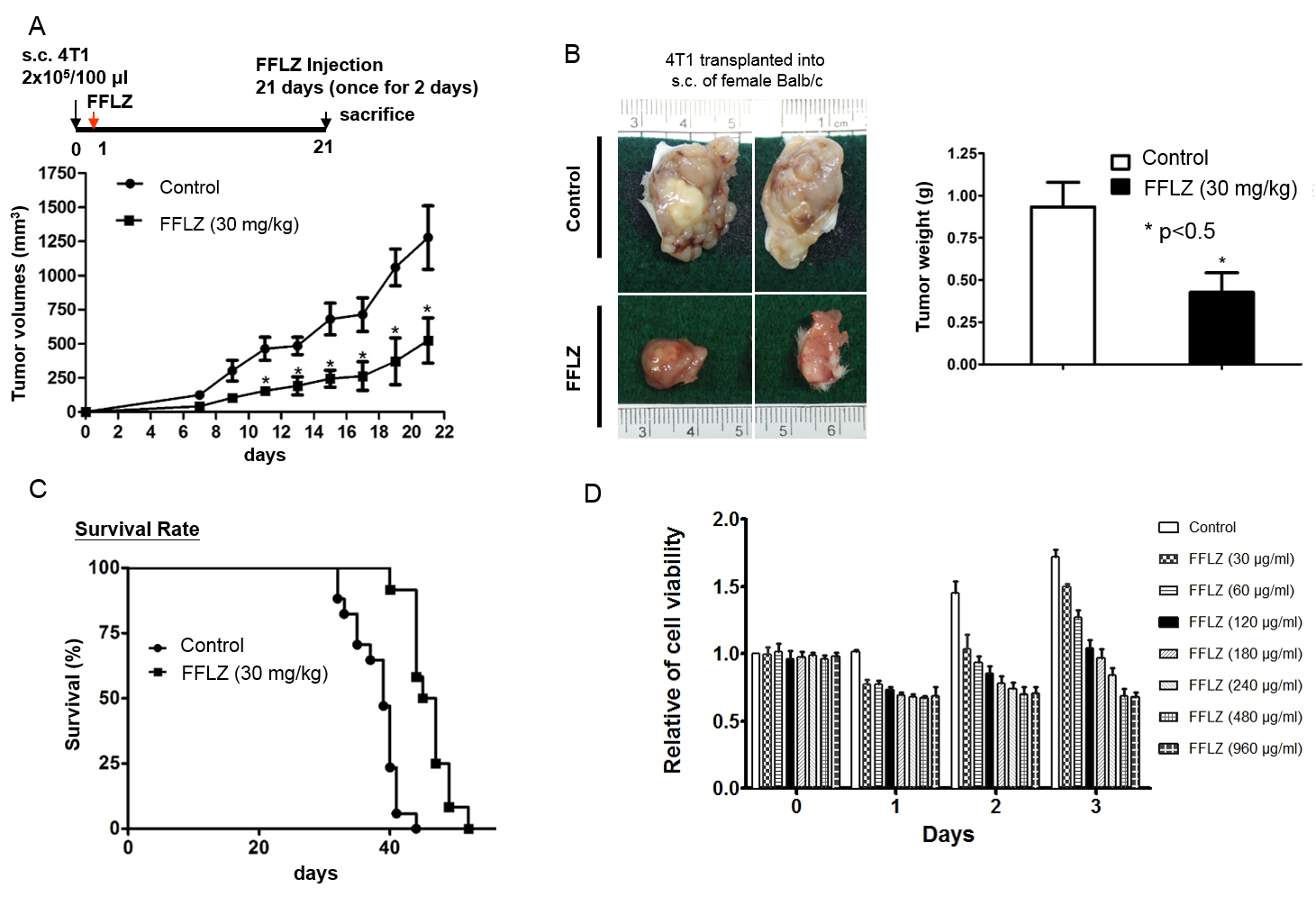


## Sup. Figure 1. Fucose-containing fraction of Ling-Zhi (FFLZ) inhibits carcinogenesis *in vivo*.

**A.** FFLZ inhibits tumor growth in 4T1-bearing mice. 4T1 (2 × 10^5^ cells) were subcutaneously (s.c.) implantated into mice (n = 6 in each group). After 1 day, mice were treated with FFLZ (30 mg/kg) Intraperitoneal (i.p.) injection at intervals of 2 days for 21 days. The tumor volume was measured at different time-points after inoculation with breast cancer cells. Data shown are the mean ± standard deviation from three independent experiments.

**B.** Pictures of tumors from mice and quantitation of the tumor weight.

**C.** FFLZ treatment increased the survival rate of mice (n = 6). Survival of control (n = 6) and FFLZ (n = 6) mice inoculated with 4T1 (2 × 10^5^ cells) through the s.c. transplantation. Data shown are the mean ± standard deviation from three independent experiments.

**D.** MDA-MB-231 cells (1×10^4^/well in 96-well plate) were treated with various dosages of FFLZ (30-960 μg/ml) for 24 and 48 h as indicated, and cell viability was determined by MTT assay. Each group at 30-960 μg/ml of FFLZ is normalized to the un-treated control. The data are representative of the mean ± SD of three separate experiments; error bars indicate SD.


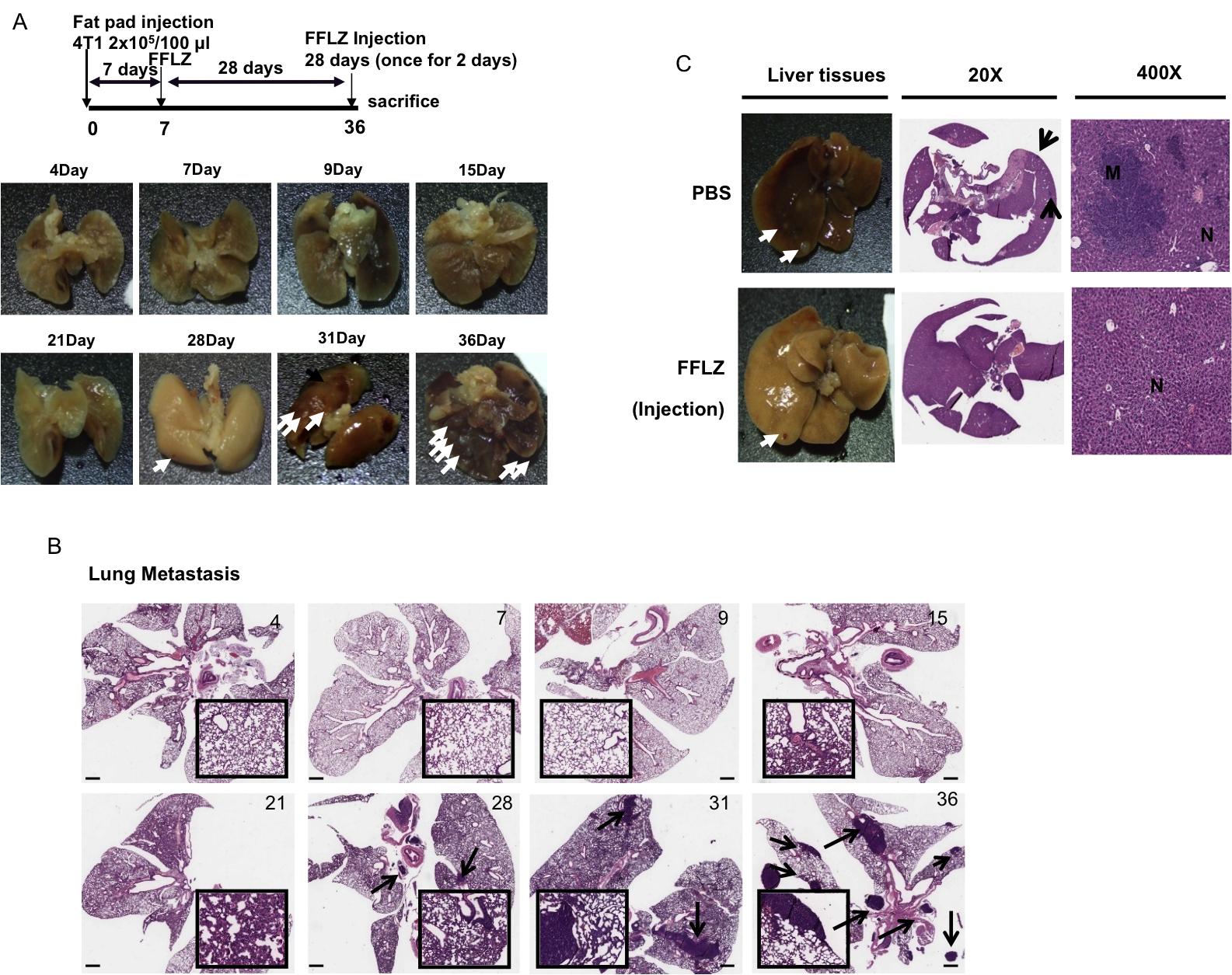


## Sup. Figure 2. FFLZ inhibits spontaneous pulmonary metastasis in mouse lungs and liver and changes the morphology of breast cancer cells.

**A.** Post treatment: FFLZ inhibits tumor growth in 4T1-bearing mice. 4T1 cells (2 × 10^5^) were implanted into mouse mammary fat pad tissue (n = 6 in each group). After 7 days, mice were treated with FFLZ (30 mg/kg) by i.p. injection at intervals of 2 days for 36 days.

**B.** Pictures and hematoxylin and eosin staining from lung lesions.

**C.** Pictures and hematoxylin and eosin staining from liver lesions.


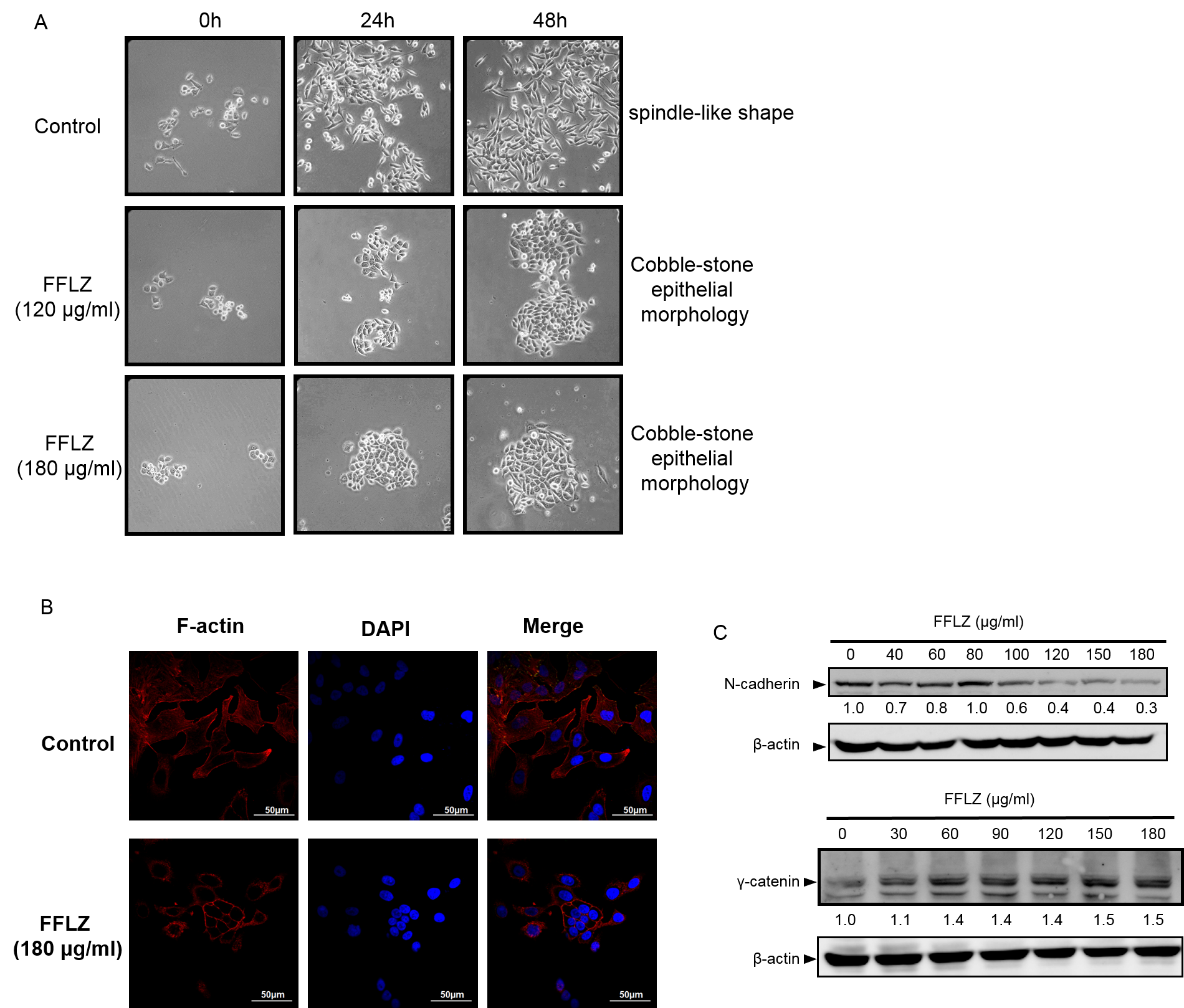


## Sup. Figure 3. FFLZ modulates the morphology of breast cancer cells and the expression of EMT markers.

**A.** Morphology of MDA-MB-231 cells observed under microscope (0 - 48 h).

**B.** Immunofluorescence showing the morphology and actin organization (focal adhesion) of MDA-MB-231 cells treated with FFLZ (200 μg/ml) for 24 h. The red color indicates F-actin stained with anti-phalloidin antibody, and the blue color indicates nuclei stained with DAPI. Scale bar = 20 μm.

**C.** Total protein lysates derived from 4T1 cells were treated with FFLZ for 48 h. Lysates were subjected to western blot analysis for determination of protein expression of the indicated EMT markers, N-cadherin and γ-cadherin.


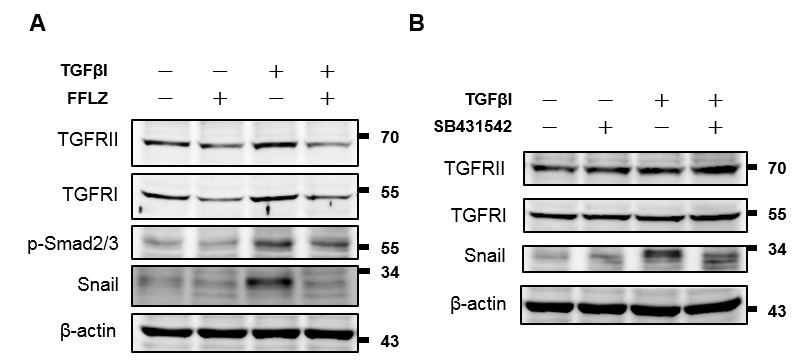


## Sup. Figure 4. FFLZ down-regulates the TGFβ1-induced phosphorylation of Smad2/3 and expression of Snail

**A.** Total protein lysates derived from MDA-MB-231 cells pre-treated with TGFβ1 (1 ng/ml) for 0.5 h and then treated with FFLZ (400 μg/ml) for 4 h.

**B.** Total protein lysates derived from MDA-MB-231 cells pre-treated with TGFβ1 (1 ng/ml) for 0.5 h and then treated with SB431542 (10 μm) for 4 h. The indicated proteins were detected by western blot analysis.


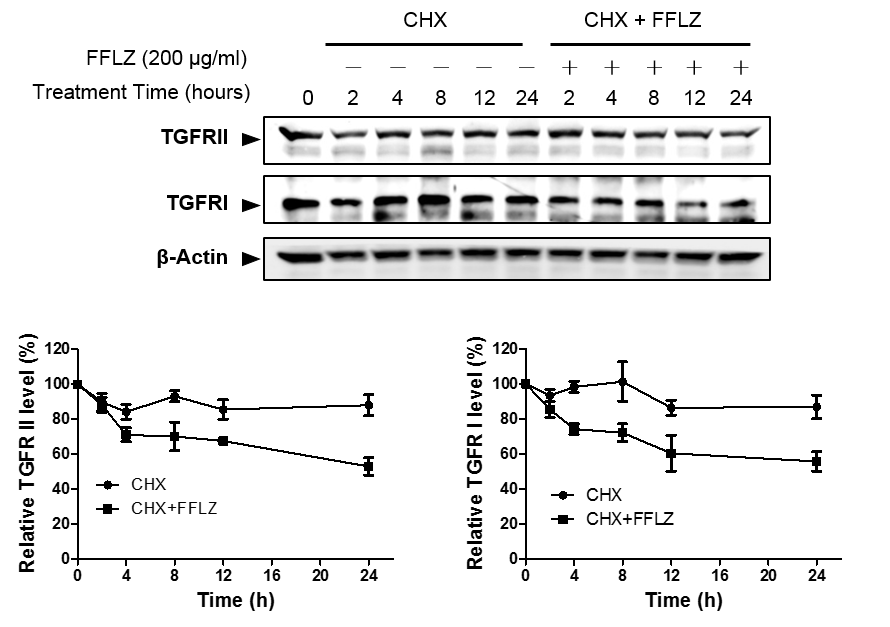


## Sup. Figure 5. FFLZ down-regulates TGFβ receptors via accelerated ubiquitin-dependent proteasome-mediated degradation in 4T1 breast cancer cells.

Time course of TGFRI and TGFRII degradation after addition of cycloheximide (CHX, 10 μg/ml) in the presence and absence of FFLZ (200 μg/ml) for 0 to 24 h in 4T1 cells. Quantification of TGFRs band intensities in an experiment, representative of three separate determinations by ImageJ.


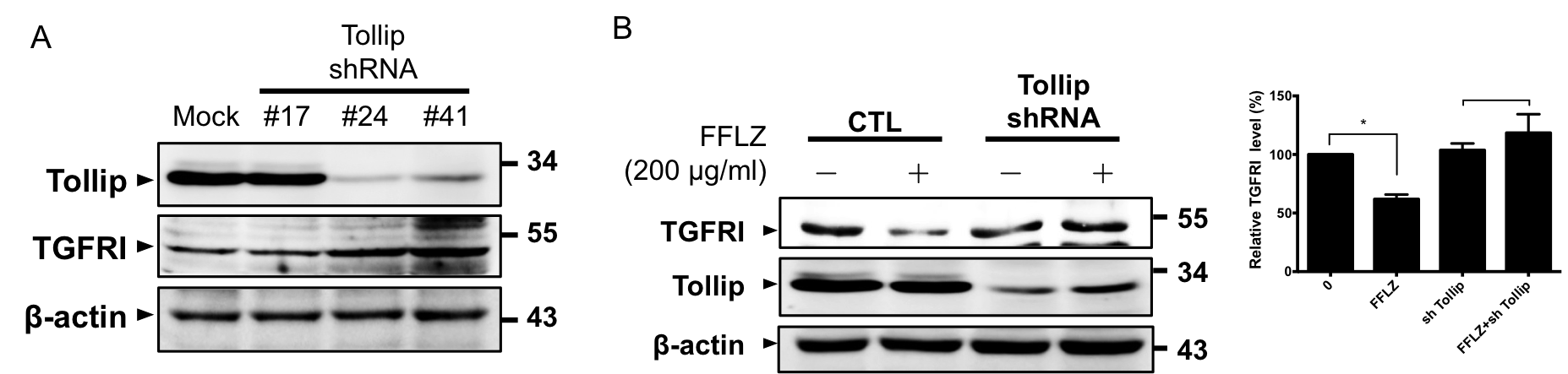


## Sup. Figure 6. FFLZ reduces Tollip binding to TGFRI to modulate ubiquitin-dependent proteasome-mediated degradation.

**A.** MDA-MB-231 cells transfected with mock plasmid or three shRNAs as previously described, followed by western blotting of the whole cell lysates to detect the expression of Tollip and TGFRI. β-actin was used as the internal control.

**B.** MDA-MB-231 cells (mock and knockdown) were incubated with FFLZ (200 μg/ml) for 3 h, followed by western blotting of the whole cell lysates to detect the expression of TGFRI and Tollip. β-actin was used as the internal control. Data are represented as the mean ± SD.


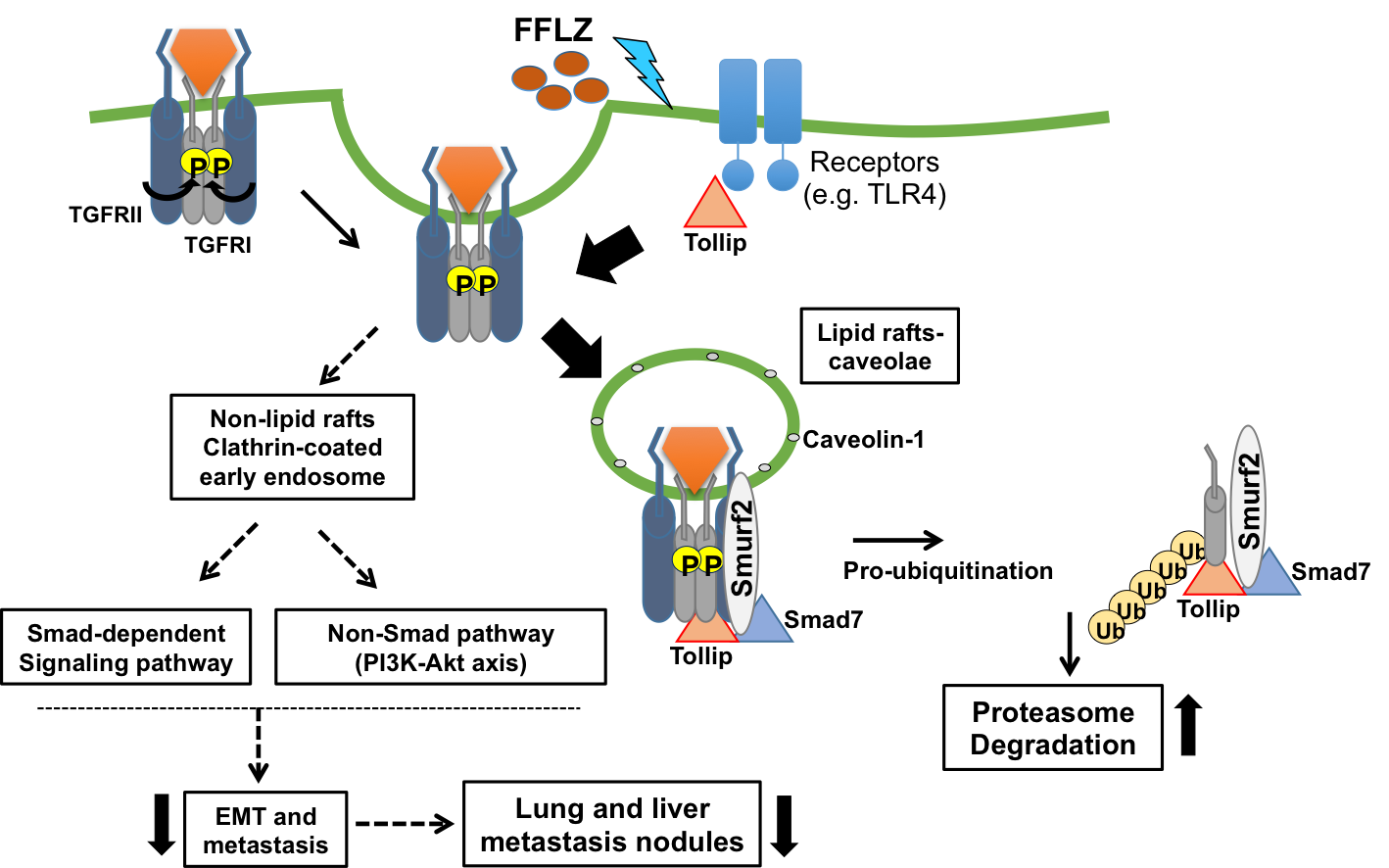


## Sup. Figure 7. Proposed scheme of FFLZ-enhanced TGFR degradation via lipid rafts-dependent ubiquitination.
